# Probing buried recombination pathways in perovskite structures using 3D photoluminescence tomography†
†Electronic supplementary information (ESI) available. See DOI: 10.1039/c8ee00928g


**DOI:** 10.1039/c8ee00928g

**Published:** 2018-08-23

**Authors:** Camille Stavrakas, Ayan A. Zhumekenov, Roberto Brenes, Mojtaba Abdi-Jalebi, Vladimir Bulović, Osman M. Bakr, Edward S. Barnard, Samuel D. Stranks

**Affiliations:** a Cavendish Laboratory , JJ Thomson Avenue , Cambridge CB3 0HE , UK . Email: sds65@cam.ac.uk; b Division of Physical Sciences and Engineering , King Abdullah University of Science and Technology (KAUST) , Thuwal 23955-6900 , Kingdom of Saudi Arabia; c Research Laboratory of Electronics , Massachusetts Institute of Technology , 77 Massachusetts Avenue , Cambridge , MA 02139 , USA; d Molecular Foundry , Lawrence Berkeley National Laboratory , Berkeley , CA , USA

## Abstract

Perovskite solar cells and light-emission devices are yet to achieve their full potential owing in part to microscale inhomogeneities and defects that act as non-radiative loss pathways. These sites have been revealed using local photoluminescence mapping techniques but the short absorption depth of photons with energies above the bandgap means that conventional one-photon excitation primarily probes the surface recombination. Here, we use two-photon time-resolved confocal photoluminescence microscopy to explore the surface and bulk recombination properties of methylammonium lead halide perovskite structures. By acquiring 2D maps at different depths, we form 3D photoluminescence tomography images to visualise the charge carrier recombination kinetics. The technique unveils buried recombination pathways in both thin film and micro-crystal structures that aren’t captured in conventional one-photon mapping experiments. Specifically, we reveal that light-induced passivation approaches are primarily surface-sensitive and that nominal single crystals still contain heterogeneous defects that impact charge-carrier recombination. Our work opens a new route to sensitively probe defects and associated non-radiative processes in perovskites, highlighting additional loss pathways in these materials that will need to be addressed through improved sample processing or passivation treatments.

## Abstract 2

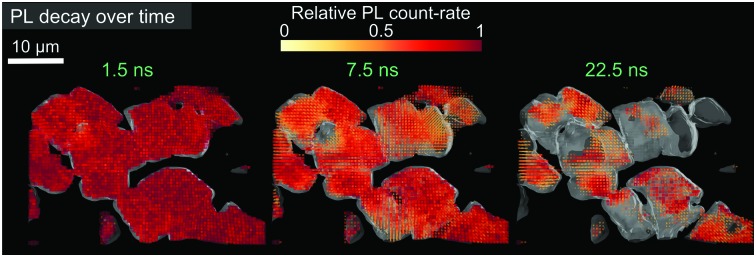
Perovskite solar cells and light-emission devices are yet to achieve their full potential owing in part to spatially heterogeneous non-radiative loss pathways that are both on, and buried beneath, the surfaces of films and crystals.

##

Broader context

Halide perovskites are generating enormous excitement for use in photovoltaics and LEDs. However, even the best perovskite devices contain defects which trap energised electrons and thus limit performance. One way to probe the presence of defects in a semiconductor is to generate energised electrons through illumination (*cf.* a solar cell) and monitor the luminescence as the electrons relax back to the ground state. The defects provide a competing pathway in which the electrons lose their energy to heat instead of by emitting photons. Here, we probe luminescence deep into the film using an analogous approach to that used in biological imaging. Two-photon excitation allows us to acquire luminescence maps at any depth in the sample, and then use the stack of maps to generate a 3D image of the luminescence. We find that state-of-the-art perovskite thin films and even single crystals are very spatially heterogeneous in luminescence and thus defect distributions, both laterally and in depth. We also show that defect ‘healing’ approaches using illumination primarily impact the surface defects but not deeper defects. This imaging technique is a powerful, sensitive defect probe, allowing us to monitor the efficacy of passivation approaches which will ultimately take inexpensive perovskite devices to their performance limits.

## Introduction

Since a key breakthrough in 2012,^1^,^2^ the power conversion efficiency of perovskite photovoltaic (PV) solar cells and light-emission devices (LED) has risen dramatically to 22%^3^ and 8–12%^4^–^6^ respectively, with the efficiency of laboratory PV cells now surpassing the commercial thin film PV technology cadmium telluride (CdTe).^7^ Such rapid progress, combined with low-cost fabrication and band-gap tunability,^8^ has motivated the use of these materials in the fabrication of high-performance devices,^9^ along with efforts to understand the mechanisms underpinning and limiting their radiative efficiency.^10^

Solution-processed films can be fabricated rapidly, showing great promise for large-scale manufacturing^11^ and printable optoelectronic devices.^12^ Single crystals can also be grown in millimetre size and as bulk films of interconnected micro-crystals.^13^,^14^ Despite high defect levels of the order of 10^15^ cm^–3^ in polycrystalline films, a million times higher than in gallium arsenide (GaAs) or silicon (Si), polycrystalline perovskite devices still operate remarkably well.^9^ Nevertheless, these defects are yet not entirely benign, and lead to substantial fractions of non-radiative decay which still dominates recombination of charge carriers in perovskite structures under solar illumination conditions.^15^ For example, recent reports of photoluminescence maps have revealed a grain-to-grain^16^ heterogeneity in the emission from surfaces of metal-halide perovskite polycrystalline films, which leads to substantial losses in PV and LED devices.^17^–^19^ However, these maps are dominated by photoluminescence from the surface of the films due to the finite absorption depth of the photons directly photo-exciting the material, and thus we do not gather information about important local recombination pathways buried beneath the surface.

Extensively used in biology for imaging over the past 20 years,^20^–^22^ two-photon photoluminescence (2P-PL) spectroscopy is based on the near-simultaneous absorption of two photons with an energy lower than the materials’ band-gap. This is a non-linear optical process that only occurs in a measurable quantity at high excitation intensities compared with one-photon photoluminescence (1P-PL). As such, absorption only occurs at the focal point of the microscope where the local concentration of photons is sufficiently high. At lower intensities outside of the focal point, the material is transparent to these photons due to the sub-bandgap photon energy. This allows for 3D optical sectioning of the sample, while avoiding excitation of the sample out of the desired focal plane. Time-resolved two-photon confocal photoluminescence (2P-TRPL) was recently developed to probe carrier lifetimes in semiconductors,^23^ and was used successfully to investigate the passivation of CdTe thin film solar cells.^24^ Strong two-photon absorption in perovskites has been reported^25^–^27^ and macroscopic 2PPL measurements revealed the existence of different recombination pathways in the bulk and at the surface of films.^28^,^29^ 2P-TRPL has also been used to investigate carrier recombination in perovskite single crystals,^30^,^31^ quantum dots^32^ and microplates.^33^ Three-dimensional tomography has been performed on perovskite crystals using one-photon excitation but the strong absorption coefficient and finite absorption depth^34^ means that optical effects such as those at the edges dominate and mask crucial information about the bulk.^35^

In this communication, we perform 2P-TRPL microscopy on methylammonium lead iodide (MAPbI_3_) and bromide (MAPbBr_3_) polycrystalline and micro-crystal films to compare local surface and bulk recombination properties. We reveal buried recombination pathways in both polycrystalline and micro-crystal films. We also demonstrate the technique on photo-brightened (passivated) films to show that such approaches primarily passivate the surfaces and additional work will be required to passivate the non-radiative pathways below the surface. For the first time, we demonstrate time-resolved 3D tomography on microcrystal films of MAPbBr_3_ and MAPbI_3_ and form 4D images of the PL with temporal and spatial resolution. We again reveal buried, heterogeneous recombination pathways even in structures that have been previously considered as single crystals. Our work opens up 4D spectroscopy for the exploration of optical properties and recombination pathways in perovskites films, crystals and full device structures.

## Results and discussion

**Fig. 1:**
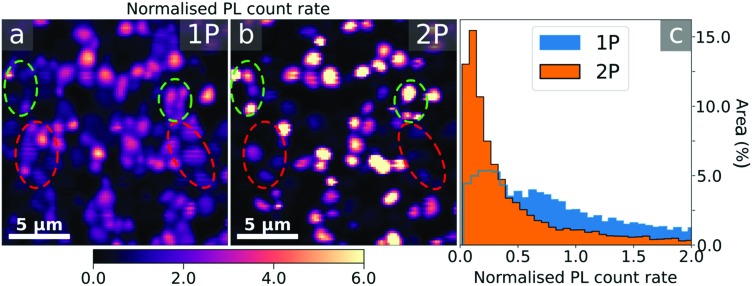
2D photoluminescence (PL) maps of a MAPbI_3_ film normalised to their respective mean using (a) one-photon (1P-PL) and (b) two-photon (2P-PL) excitation at 510 nm and 1100 nm respectively, with a pulsed (5 MHz, pulse width 150 fs) excitation density of ∼10^16^ cm^–3^. (c) Comparison of the PL distributions from the two maps, normalised by the mean of each. The red dotted circles in (a) and (b) highlight grain clusters that are bright at the surface and dark in the bulk; the green dotted circles show the opposite trend.

We solution-processed thin films of MAPbI_3_ on cover slip glass following previous methods,^16^,^36^,^37^ which yield high-performing photovoltaic devices when prepared in a full device stack (see Fig. S1 (ESI†) for sample characterisation). In Fig. 1a, we show a confocal PL intensity map using direct (one-photon, 1P) excitation with pulsed illumination at 510 nm (2.43 eV, 150 fs pulse width) to selectively generate excitations nearer to the surface than the bulk. This reveals grain-to-grain heterogeneity in luminescence,^16^,^18^,^19^ which at the low excitation densities used here (∼10^16^ cm^–3^) arises primarily from trap-limited heterogeneous recombination rather than local diffusion effects.^19^ In Fig. 1b, we show the PL intensity map of the same scan region but impinging on the sample sub-bandgap photon pulses (1.27 eV, wavelength 1100 nm, 150 fs pulse width) below the bandgap of the perovskite (1.6 eV), giving rise to two-photon (2P) excitation in the bulk of the film. The excitation fluences used were tuned to obtain similar total PL count rates for both 1P and 2P measurements, which yield comparable charge excitation densities in both cases. We note that a precise calculation of charge density would require complex optical modelling and knowledge of the 2P absorption cross-section, but this provides an approximate matching for comparison purposes. We estimate the waist of the focused excitation beam to be such that 2-photon absorption occurs through the entire thickness (∼350 nm) of the films, and hence we are comparing surface-dominated excitation (1P) with bulk-dominated excitation (see Fig. S2 and S3 in ESI† for further discussion). We normalise the counts of both maps to the mean counts of each map to allow a relative comparison between the maps (see similar results when normalising to the maximum counts in Fig. S4, ESI†). We find that some grains, such as those circled in red in Fig. 1a and b, are bright on the surface but relatively dimmer in the bulk. We observe the opposite trend for some other grains (for example the clusters in green dotted circles), while many of the others are similarly bright or dark on the surface and in the bulk. From these maps, it is also clear that the contrast between dark and bright grains is greater when using 2P excitation. This could be because local diffusion, which can act to homogenise the local emission heterogeneity, could potentially be more difficult through grain boundaries in the bulk compared to at the surface.^19^ In Fig. 1c, we compare the relative luminescence for the 1P and 2P maps, with each again normalised to the mean counts for the maps. We find a tighter distribution of the 2P map with a long tail of brighter grains suggesting that the emission is closer to a normal (Gaussian) intensity distribution on the surface (1P) while being more localised below it (2P). This is even more exaggerated in state-of-the-art alloyed triple cation samples (Cs_0.06_FA_0.79_MA_0.15_)Pb(I_0.85_Br_0.15_)_3_ (see Fig. S6 and S7 (ESI†) for sample and device characterisation). We note that the spectrally-resolved PL maps (Fig. S5, ESI†) show very little difference between 1P and 2P excitation. These results collectively reveal new buried non-radiative recombination pathways that will need to be addressed to push even the highest performing devices to their radiative limits.

**Fig. 2:**
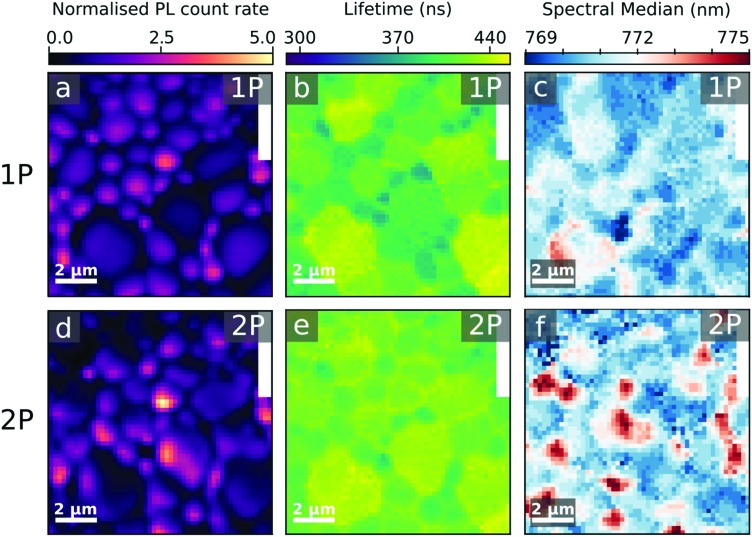
PL maps of a MAPbI_3_ film after light soaking in humid air (30 minutes under 532 nm laser illumination at charge densities equivalent to ∼2 sun in air at 45% relative humidity). (a and d) PL count rate maps (normalised to their respective mean) acquired using (a) 1P (510 nm) and (d) 2P (1100 nm) excitation with a pulsed (1.25 MHz, 150 fs pulse width) excitation density of ∼10^16^ cm^–3^. (b and e) Lifetime maps extracted from (b) 1P- and (e) 2P-TRPL measurements. The lifetime is defined as the time at which 63% of the photons have been detected. (c and f) Spectral median maps extracted from 1P-PL and 2P-PL spectra. The regions marked in white denote marker particles on the surface of the film and were excluded from the analyses.

We and others recently found that light and atmospheric treatments^36^,^38^–^40^ on polycrystalline MAPbI_3_ perovskite thin films can result in large enhancements in the luminescence. These studies have proposed that the surfaces are selectively passivated though this has not yet been directly shown. Here, we use 1P- and 2P-TRPL to image the surface and the bulk of a MAPbI_3_ film following an *ex situ* light soaking treatment in humid air, with illumination from a 532 nm laser in 45% relative humidity for 30 minutes under intensities generating charge densities equivalent to ∼2 suns. In Fig. 2a and d we show PL count rate maps obtained using 1P- and 2P-TRPL, respectively (see Fig. S8 (ESI†) for an equivalent untreated film). As before, the 1P and 2P excitation fluences were chosen in order to obtain comparable PL count rates. Both 1P and 2P count rate maps, normalised to their mean, show that the PL is significantly more uniform in the treated sample, with the greatest relative enhancement observed at the surface with 1P excitation (Fig. 2a and Fig. S9, ESI†).

**Fig. 3:**
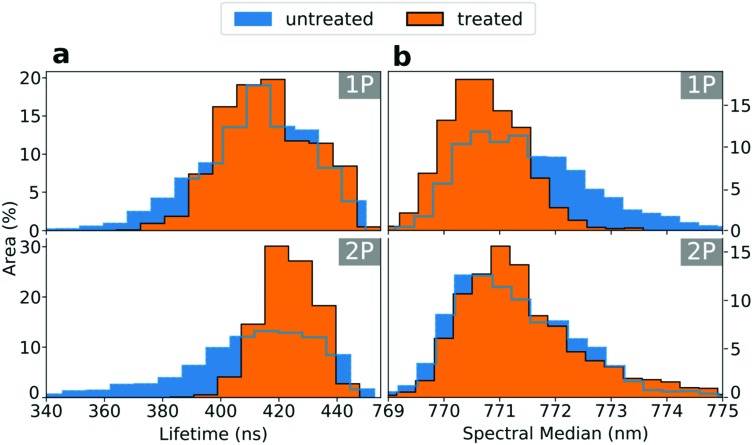
Comparison between (a) PL lifetime and (b) PL spectral median distributions in the untreated (blue) and treated (orange) films, extracted from the 2D maps in Fig. 2 and Fig. S8 (ESI†) using one-photon (1P) and two-photon (2P) excitation.

We plot the PL lifetime maps in Fig. 2b and e and compare the PL lifetimes for the treated and untreated samples in Fig. 3a. Here, we define lifetime as the time at which 1 – 1/*e* (∼63%) of the total photons have been detected following the excitation pulse, hence accounting for both the initial fast decay and the long-lived emission. Despite the increased homogeneity in emission in 1P-PL we observe a lengthening of the 2P-TRPL lifetime compared to the 1P-TRPL, which is particularly significant for the treated sample (Fig. 3a). Interestingly, for both 1P- and 2P-TRPL we observe a positive correlation between the PL lifetime and grain PL in the untreated film, but this turns into an anti-correlation in the treated film (Fig. S10, ESI†). We ascribe this observation to a decrease in the trap density after treatment, particularly at the surface,^16^,^36^ which changes the nature of the primary recombination pathway for the same excitation fluence from trap-assisted (lifetime increases with decreasing trap density, *i.e.* with darker grains) to bimolecular (lifetime decreases with decreasing trap density, *i.e.* with brighter grains).^18^,^41^ The longer 2P-TRPL lifetimes compared to the 1P-TRPL (Fig. 3b), particularly for the treated sample which follows bimolecular recombination kinetics, suggests that the treatment is most effective at the surface, leaving the bulk more defective than the surface. We note that the same grains appearing bright at the surface are also bright in the bulk (Fig. S11, ESI†). We also note that these observations on carrier lifetimes could be further complicated by any changes to local photon recycling due to increased luminescence yields,^42^ which would selectively increase the bulk lifetime more than the surface lifetime, or potential changes to local carrier diffusion^19^ in the bulk and/or at the surface.

In Fig. 2c and f we show the maps of the spectral median across the treated film, using 1P and 2P excitation, respectively (see Fig. S8 (ESI†) for the untreated film). We show a direct comparison of the spectral median distributions for the treated (orange) and untreated (blue) films in Fig. 3b, unveiling a blueshift of the luminescence from the surface (1P) of the treated film. This could be consistent with the treatment leading to a reduction in shallow trap density (band-tail states),^16^,^18^,^43^ leading to slightly higher energy emission. It may also be consistent with a greater fraction of the emission coming from a well-passivated surface than the bulk; emission from the latter would be red-shifted with respect to the surface through photon reabsorption and re-emission.^42^ We note that some grains show red-shifted 2P-PL emission in both the treated (Fig. 2f) and untreated (Fig. S8, ESI†) samples, which do not correlate with PL count-rate or lifetime on these specific grains; these could also be due to reabsorption events, although it is not yet clear why we observe them at sites that aren’t necessarily more emissive than others.

**Fig. 4:**
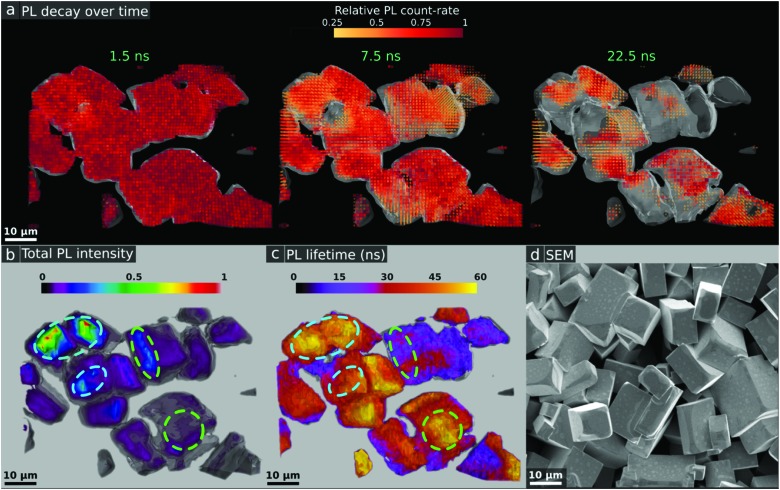
Time-resolved 3D tomography PL images of a micro-crystal film of MAPbBr_3_ using 2P-TRPL with a resolution of ∼1 μm in depth and 2P excitation fluence of ∼6000 μJ cm^–2^. The greyscale topography background is a PL isosurface that represents the surface of the crystals. (a) Snapshots showing the relative spatial PL decay over time following the excitation pulse. For clarity, the lower quartile of the relative PL count rate is not shown. These images can be directly compared with the (b) total PL intensity and the (c) extracted lifetime, with both images showing some pockets of strong PL associated with long lifetime (blue circles) and some showing the opposite behaviour (green circles). (d) Representative SEM image of the crystal films.

Finally, we demonstrate the power of two-photon photoluminescence microscopy by constructing 3D PL tomography images of micro-crystal films of MAPbBr_3_ (Fig. 4) shown in the SEM image in Fig. 4d (see Fig. S12 (ESI†) for X-Ray Diffraction (XRD) pattern) and MAPbI_3_ (Fig. S13, ESI†). We record a series of 2D 2P-TRPL maps, moving the focal point of the laser further into the bulk with a step of 1 μm between each map. In Fig. 4a, we show a time series of snapshots to visualize the PL decay over time after the excitation pulse (see ESI† for Video). We note that for clarity we only show pixels with a relative PL count rate above 25% (see Fig. S14, ESI† for a visualisation of the full dataset). From the PL count rate data we plot isosurfaces, yielding the 3D image of the PL emission within single crystals shown in Fig. 4b. In the same way, we display the 3D tomography of the lifetime in Fig. 4c. We note that for the time-resolved measurements we use an optical long-pass filter to only collect photons in the longest wavelength tail of the emission to minimise the impact of reabsorption effects, which may otherwise cause us to probe different emitting species at different depths.^24^ In Fig. 4b and c, we compare the PL count rate and lifetime, and find that we again observe buried recombination and heterogeneity even within these structures which have been nominally reported as single crystals.^14^ Interestingly, some pockets of strong luminescence intensity are associated with long lifetimes (blue circles in Fig. 4b and c) while some other bright regions are not (green circles). By performing fluence-dependent measurements through a depth slice of one of the micro-crystals (Fig. S15 and S16, ESI†), we find that a bright region shows a decreasing lifetime with increasing excitation fluence, which is consistent with a radiative bimolecular recombination regime.^41^ By contrast, the dark regions show an increasing lifetime with increasing excitation fluence, consistent with a trap-limited recombination regime in which a high density of traps become filled with carriers at higher charge density.^44^ In fact, our results are consistent with these micro-crystal samples having a non-negligible density of defects that may have a large impact on device performance,^45^ and therefore will be crucial to understand and eliminate. We note that a quantitative trap density analysis would require complex optical modelling including effects such as interference, surface roughness, and refraction. Future work would be required to reconcile these trap densities with those reported previously using space-charge-limited current methods.^46^,^47^ This luminescence tomography approach will be particularly powerful because it is sensitive to even low densities of defects or low bandgap emission sites and therefore will detect inhomogeneities that might be beyond the resolution of conventional analytical techniques such as XRD, electroluminescence or wide field 1P-PL.

## Conclusion

In summary, we have used time-resolved two-photon microscopy (2P-TRPL) as a four-dimensional (4D) imaging technique to explore buried recombination pathways in perovskite structures. We find stark differences between 1P (surface-dominant) and 2P (bulk-dominant) luminescence in MAPbI_3_ thin films, with some grains showing bright luminescence from the surface but weak luminescence, and some others showing the opposite behaviour. We increase the differences by considering light-induced passivated treatments, revealing that the surfaces are more selectively passivated than the bulk. We constructed for the first time time-resolved 3D PL images of MAPbI_3_ and MAPbBr_3_ micro-crystals, revealing substantial luminescence inhomogeneities even in structures which are nominally single crystals. Our work highlights the power of 2P-TRPL mapping as a sensitive 4D probe for defects both on the surface as well as buried beneath the surfaces and at interfaces. Understanding and eliminating these loss pathways will be crucial for further development of perovskite PV solar cells and LED devices.

## Conflicts of interest

There are no conflicts to declare.

## Supplementary Material

Supplementary information

Supplementary movie
