# Supplementary material for: Probing buried recombination pathways in perovskite structures using 3D photoluminescence tomography
Source: Energy Environ Sci. 2018 Aug 23;11(10):2846–52. doi: 10.1039/c8ee00928g (PMC6333269; doi:10.1039/c8ee00928g)
Supplement: Supplementary file 1 [file EE-011-C8EE00928G-s001.pdf]

Electronic Supplementary Material (ESI) for Energy & Environmental Science.
This journal is © The Royal Society of Chemistry 2018

Electronic Supplementary Information

Probing Buried Recombination Pathways in Perovskite Structures using 3D Photoluminescence Tomography

*Camille Stavrakas[1], Ayan A. Zhumekenov[2], Roberto Brenes[3], Mojtaba Abdi-Jalebi[1], Vladimir Bulovic[3], Osman M. Bakr[2], Edward S. Barnard[4], Samuel D. Stranks[1]**

[1]Cavendish Laboratory, JJ Thomson Avenue, Cambridge CB3 0HE, United Kingdom

[2]Division of Physical Sciences and Engineering, King Abdullah University of Science and Technology (KAUST), Thuwal 23955-6900, Kingdom of Saudi Arabia

[3]Research Laboratory of Electronics, Massachusetts Institute of Technology, 77 Massachusetts Avenue, Cambridge, MA 02139, USA

[4]Molecular Foundry, Lawrence Berkeley National Laboratory, Berkeley, CA, USA;

Corresponding Author

*sds65@cam.ac.uk

Sample preparation:

MAPbI3 Samples:

Glass substrates were washed sequentially with soap, de-ionized water, acetone, and isopropanol, and finally treated under oxygen plasma for 10 min. Thin films of $MAPbI_3$ were solution processed by employing a methylammonium iodide (MAI) and lead acetate $Pb(Ac)_2{\cdot}3H_2O$ precursor mixture with a hypophosporous acid (HPA) additive[1]. MAI (Dyesol) and $Pb(Ac)_2{\cdot}3H_2O$ (Sigma-Aldrich) were dissolved in anhydrous *N,N*-dimethylformamide (DMF) at a 3:1 molar ratio with final concentration of 37 wt % and HPA added to an HPA:Pb molar ratio of ∼11%. The precursor solution was spin coated at 2,000 rpm for 45 s in a nitrogen-filled glove box, and the substrates were then dried at room temperature for 10 min before annealing at 100°C for 5 min. All samples were then stored in a nitrogen-filled glove box until used.

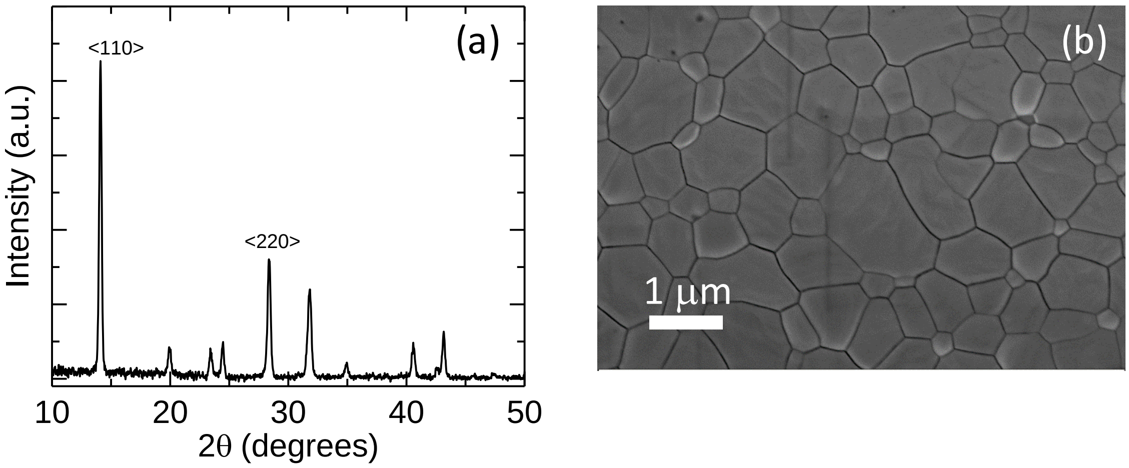

Figure S1. (a) X-Ray-Diffraction (XRD) pattern and (b) Scanning electron micrograph (SEM) of the $MAPbI_3$ films used in this work. These materials were previously used to fabricate solar cell devices achieving >15% power conversion efficiency (Brenes et al.[2]).

The light-soaking measurements were performed by photo-exciting perovskite films on coverslips enclosed in a custom-built flow chamber capable of flowing ultra-high purity gases in a controllably humidified form (40%–50% relative humidity). The samples were photo-excited with a 532-nm CW laser at intensities approximately equivalent to the photon fluxes of 2 sun irradiation (150 mW/cm^2) for 30 minutes. They were then sealed and shipped for measurement the following day.

Triple Cation Samples and Devices:

The organic cations were purchased from Dyesol; the lead compounds from TCI; CsI from Alfa Aesar. The triple-cation perovskite thin films ($(Cs_{0.06}FA_{0.79}MA_{0.15})Pb(I_{0.85}Br_{0.15})_3$, where MA = methylammonium, $CH_3NH_3^+$ and FA = formamidinium, $CH_3(NH_2)_2^+$), were deposited from a precursor solution containing FAI 1.0 M, PbI2 1.1 M, MABr 0.2 M and PbBr2 0.22 M in anhydrous DMF:DMSO 4:1 (v:v). Then CsI, dissolved as a 1.5 M stock solution in DMSO, was added to the mixed perovskite precursor (5% volume) to make triple-cation perovskite. The perovskite solution was spin coated in a two-steps program at 1000 and 4000 rpm for 10 and 30 s respectively. During the second step, 150 μL of chlorobenzene was poured on the spinning substrate 5 s prior to the end of the program. The substrates were then annealed at 100°C for 45 min in a nitrogen-filled glove box. The devices were fabricated following the same procedures for substrate preparation as well as deposition of both electron and hole transport layers (that is, TiO2, spiro-OMeTAD) developed in earlier work.[3].

Bulk micro-crystal films:

Micro-crystal films of $MAPbX_3$ (where X = Br, I) were prepared following slightly modified protocol of that reported by Saidaminov *et al.*[4] Namely, 0.3 M solutions of $MABr/PbBr_2$ in DMF and $2MAI/PbI_2$ in GBL were individually prepared and filtered through 0.22-μm pore-size filters. Each solution was mixed with 1,2-dicholorbenzene (DCB) in 5:4 volumetric ratio

and then transferred into a crystallization dish (Ø5 cm) containing pre-cleaned glass substrate. The crystallization dish was covered with a glass cover and then heated under continuous stirring at 500 rpm. After 30 mins of stirring at 40°C and 75°C, the micro-crystal films of $MAPbBr_3$ and $MAPbI_3$, respectively, were obtained on the glass substrates (see Figure S1 for X-Ray diffraction (XRD) and scanning electron microscope (SEM) images). Finally, the substrates were extracted from the solutions and annealed on a hot plate at 110°C for 5 mins. All procedures were performed at ambient conditions with ~55-57% relative humidity. The Scanning Electron Microscope (SEM) image of the $MAPbBr_3$ micro-crystal film was taken on FEI Quanta 600. XRD was measured using Bruker AXS D8 Advance powder diffractometer with Cu-Kα radiation.

Scanning electron microscopy:

The surface morphology of the films was examined using a field-emission scanning electron microscope (Merlin). An electron beam accelerated to 3 kV was used with an in-lens detector.

Solar-cell characterization:

Current–voltage characteristics were recorded by applying an external potential bias to the cell while recording the generated photocurrent with a digital source meter (Keithley Model 2400). The light source was a 450-W xenon lamp (Oriel) equipped with a Schott-K113 Tempax sunlight filter (Präzisions Glas & OptikGmbH) to match the emission spectrum of the lamp to the AM1.5G standard. Before each measurement, the exact light intensity was determined using a calibrated silicon reference diode equipped with an infrared cut-off filter (KG-3, Schott).

Two-photon spectroscopy:

For all 2P experiments, a Coherent Ultra II Ti:Sapphire excitation laser (700-1100 nm tunable, 150 fs pulse duration, 80 MHz) pumps a Coherent / APE OPO with output tuned to

1100 nm which in-turn is optically coupled to the input of the microscope through a 950 nm short-pass dichroic. We used a pulse-picker to bring the repetition rate of our laser down to 5 MHz and 1.25 MHz for TRPL measurements. Photoluminescence was filtered with a 945 nm short-pass filter, collected through a 100 μm pinhole and into detection optics. For spectral collection we used an Acton 2300i spectrometer with 150 groves/mm grating and an Andor iXon electron-multiplied CCD. For time-resolved collection we first spectrally-filtered the PL using a linear variable long-pass filter and then collected with a single-photon avalanche diode (Micro Photon Devices PDM series). A PicoQuant PicoHarp 300 time-correlated single-photon counting (TCSPC) system was used to record the timing data.

At 23-27 μW with a repetition rate of 1.25MHz, a laser pulse energy of ~20 pJ/pulse was used to excite (with 2P) the untreated and treated perovskite films in Figure 2 and Figure S8. This corresponds to a maximum pulse energy density (fluence) of 5 mJ·cm^{-2}, a time-averaged power density of 6.3 kW·cm^{-2}, and a peak power of approximately 16 GW·cm^{-2}. From the pulse energy density and the energy of a 1100 nm photon, the density of photons was ~2.8×10^{16} cm^{-2}. Assuming a β coefficient (Figure S2) of 5 cm·GW^{-1}, the absorption coefficient α at an average power of 23-27 μW is ~100 cm^{-1} giving a maximum number of absorbed photons per unit excitation volume of 2.8×10^{18} cm^{-3}. Given the uncertainty in the 2P absorption coefficient and through careful comparison with the 1P excitation densities, we estimate photo-generated carrier concentrations in our measurements to be ~10^{16}-10^{17} cm^{-3}. The pulse energy used for 1PPL with an average power of a few nanowatts is of the order of ~2 fJ/pulse, giving a fluence of about 0.1 μJ·cm^{-2} (and a carrier concentration of ~10^{16} cm^{-3})

Because of the high absorption coefficient of methylammonium lead halide perovskites, photons with energy above the band-gap are absorbed near the surface. In order for one-photon measurements to be as surface sensitive as possible, we used an excitation wavelength

of 510 nm for which we estimate a penetration depth[5] of the order of 100 nm. Single photons with wavelengths longer than the absorption edge do not carry enough energy to promote electrons to the conduction band and travel therefore deep into the material. Measurements in the bulk were performed with a 1100 nm excitation beam tightly focused by the confocal system described above.

The lateral resolution of the excitation is diffraction limited, with a full width at half maximum (FWHM) of 626 nm (Figure S3). Vertically, we exploit the fact that the transition probability for the 2P absorption process depends on the square of the intensity of the light beam used for the excitation, if the two photons have the same energy[6]. We estimate the depth resolution around the focal point to be about 1 μm. In order to mitigate the effect of re-absorption and photon-recycling, we use a linearly variable long-pass filter in the detection path to select the red-most tail of the PL emission spectrum for time-resolved measurements.

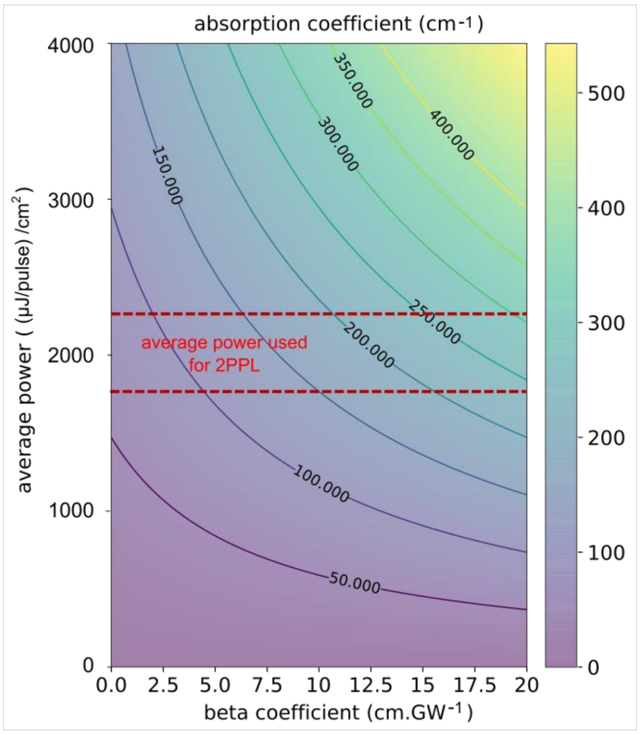

Figure S2. Parameter space for the absorption coefficient depending on the excitation power used and the value of the proportionality factor β.

The proportionality factor β between the two-photon absorption coefficient α and the light intensity was recently measured for $MAPbBr_3$ and found to be about 20 cm per GW at 800 nm[7]. At an average power of a few dozens of microwatts, the absorption coefficient at the focal point at 800 nm would be of the order of 10^3 cm^{-1}, two orders of magnitude smaller than for one-photon absorption. We expect β to be significantly lower at 1100 nm. To obtain the same order of magnitude of photoluminescence intensity, we used a few nanowatts of 1P excitation and an average power between 23 μW and 27 μW (peak power between 125 W and 145 W) for 2P. This suggests that the absorption coefficient is 3-4 orders of magnitude lower with 2P than with 1P at 1100 nm, hence somewhere around 100 cm^{-1}. By looking at the parameter space in Figure S2, β could be around 5 $cm{\cdot}GW^{-1}$, which is consistent with the fact that it should be less than 20 $cm{\cdot}GW^{-1}$, smaller at 1100 nm than its reported[7] value at 800 nm.

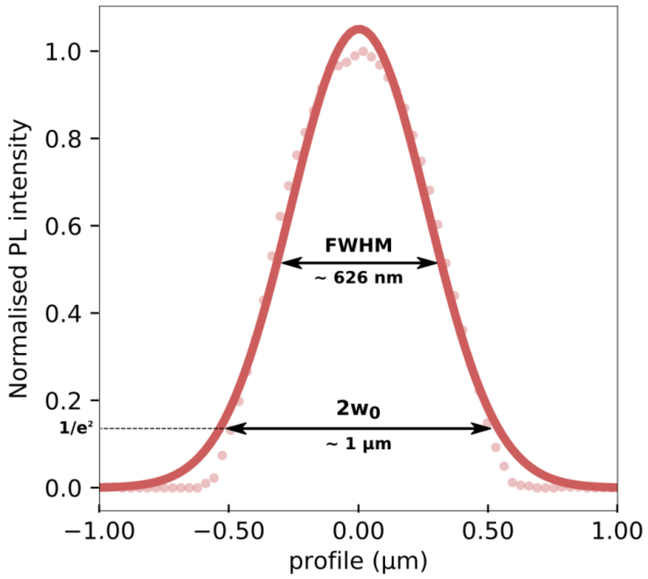

Figure S3. Beam profile of the laser used for 2P measurements.

<u>Data analysis:</u>

All maps were recorded as multidimensional matrices, and analysed using a Python code. 3D visualisation was achieved through the Mayavi module. Lifetimes were extracted from the

TRPL measurements as the time at which (1-1/e) of the photons had been detected. 3D images were reconstructed from stacks of 2D TRPL maps, from which the pixels outside of the crystals were removed.

Complementary measurements:

The 1P and 2P PL maps recorded on the $MAPbI_3$ thin film in Figure 1a and b (main text) were normalised to the mean of each distribution, respectively. Normalising to the total counts (Figure S4) shows more clearly that emission is more localised below the surface, however it is more difficult to resolve the brightness contrast between grains. Normalising to the mean highlights this contrast better. Nonetheless, comparing the two figures (with different normalisations) helps to see that the emission is a lot more homogeneous at the surface than below: the 1P map is not affected by this change in normalisation whereas the 2P map changes significantly.

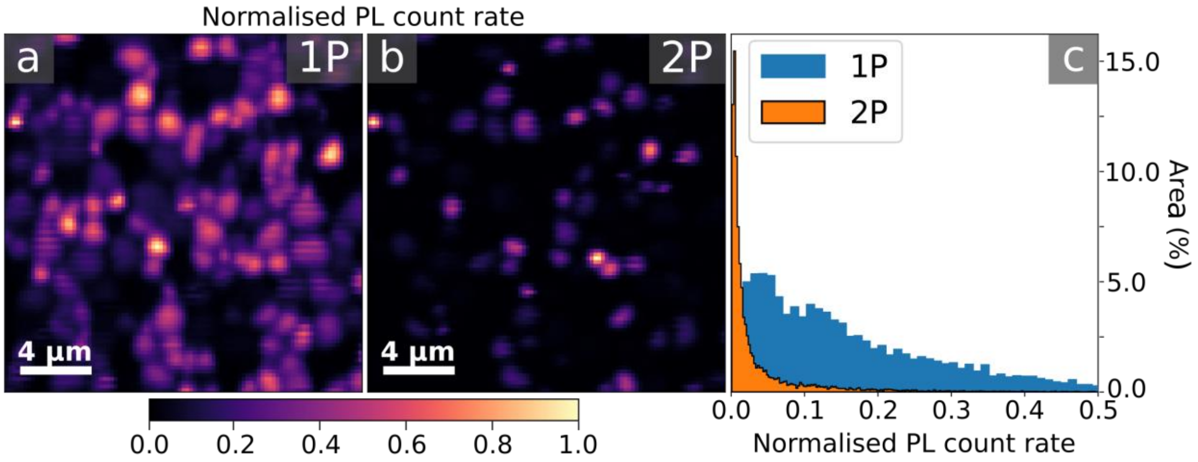

Figure S4. (a, b) 2D PL maps of the $MAPbI_3$ films from Figure 1 normalised to the total counts (instead of the mean), using one-photon (1P) and two-photon (2P) excitation. (c) Comparison of the count rate distributions again normalised to the total counts.

Spectral maps were also recorded on the $MAPbI_3$ thin film alongside with the PL maps shown in Figure 1a and b (main text) and in Figure S5a and c. In the maps in Figure S5b and d, there does not appear to be any spectral shift below the surface.

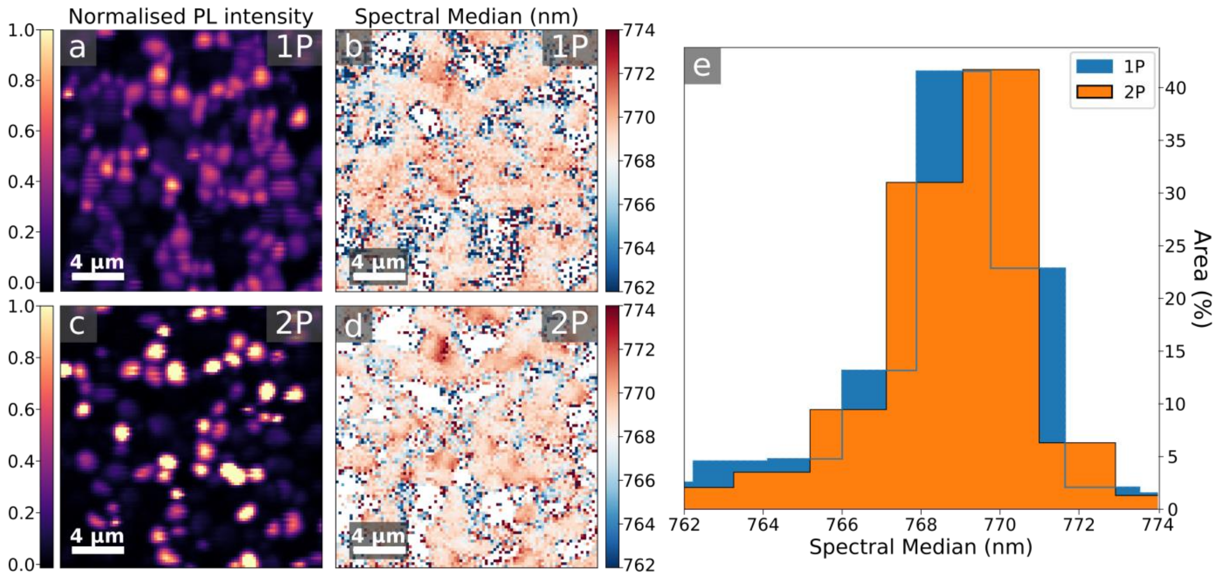

Figure S5. (a, b) 2D PL maps of the $MAPbI_3$ films from Figure 1 normalised to their respective mean between 0 and 6, using one-photon (1P) and two-photon (2P) excitation. (b,d) spectral median maps extracted from 1P-PL and 2P-PL data. (e) Comparison of the spectral median distributions.

Triple Cation Samples:

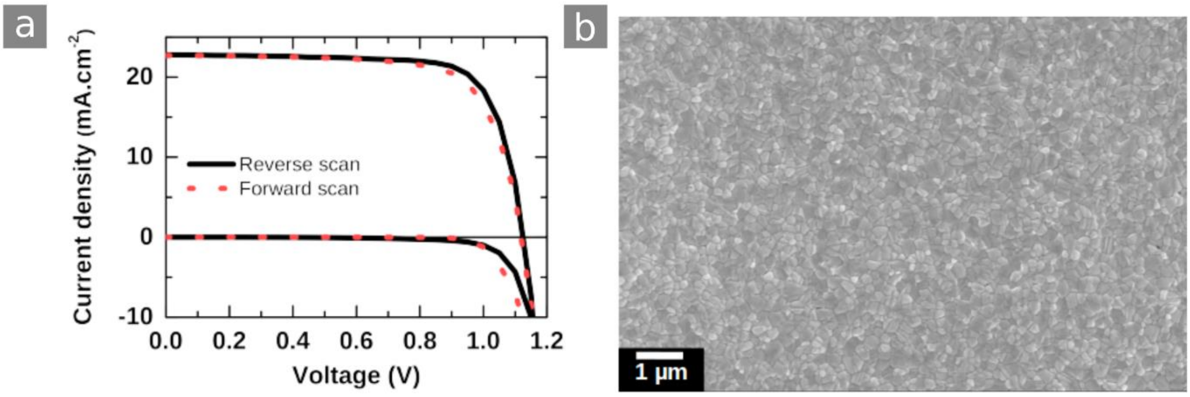

c

	J_{sc} [mA.cm^{-2}]	V_{oc} [V]	FF	PCE [%]
$(Cs,FA,MA)Pb(I_{0.85}Br_{0.15})_3$				
Reverse	22.8	1.124	0.76	19.4
Forward	22.7	1.118	0.73	18.5

Figure S6. (a) Current-voltage curves measured under AM1.5 100mW/cm^2 simulated sunlight and in the dark for the alloyed triple cation sample $(Cs_{0.06}FA_{0.79}MA_{0.15})Pb(I_{0.85}Br_{0.15})_3$ films in the full device stack FTO (fluorinated tin oxide)/c-TiO_2/meso-TiO_2/Perovskite/Spiro-OMeTAD/Au. (b) Scanning electron micrograph (SEM) of a triple cation film characterised in this work. (c) Device parameters from a (FF is fill factor, PCE is power conversion efficiency).

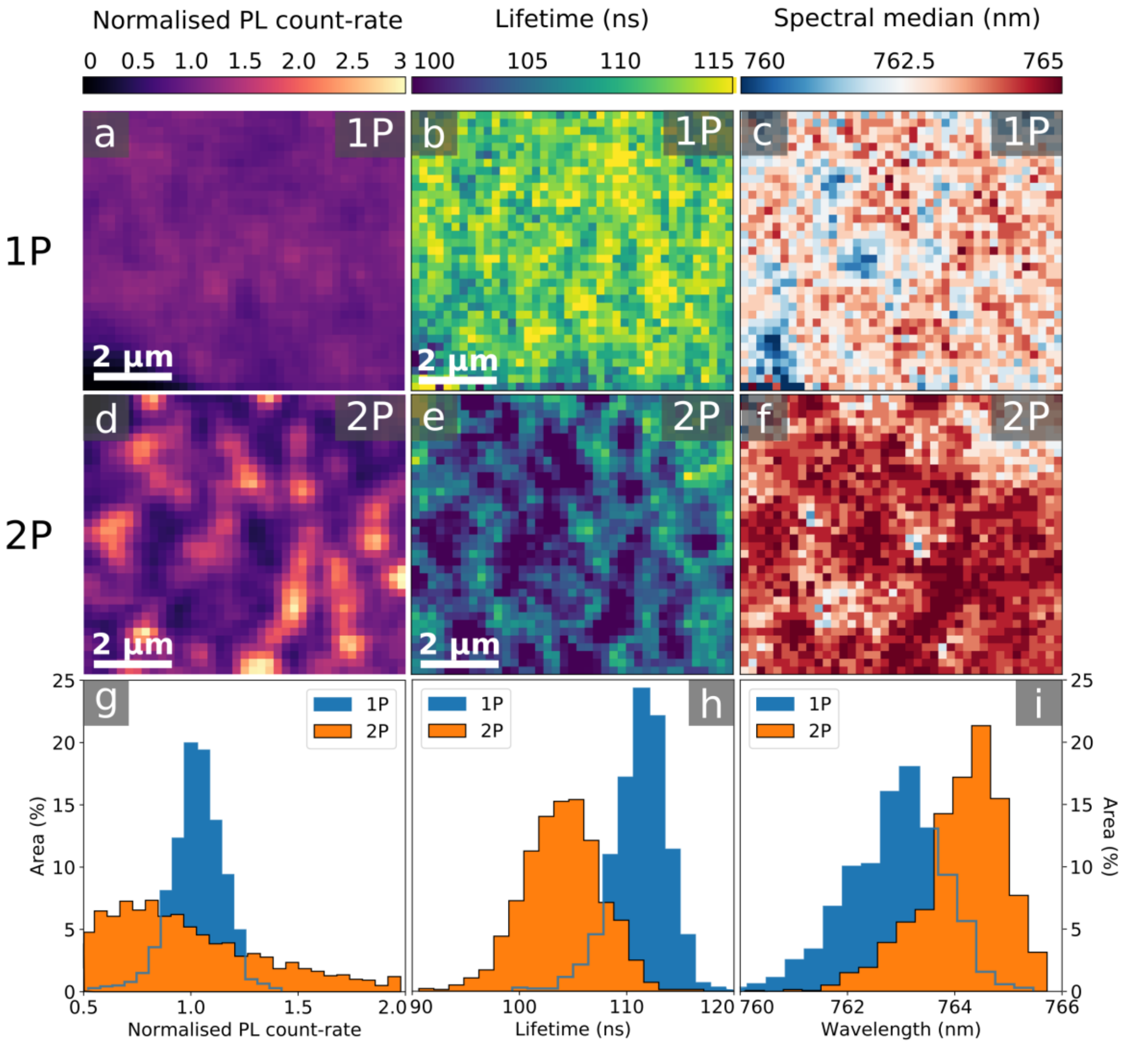

Figure S7. 1P and 2P PL count-rate (a, d, g), lifetime (b,e, h) and spectral median (c, f, i) maps and statistical distributions from a triple cation $(Cs_{0.06}FA_{0.79}MA_{0.15})Pb(I_{0.85}Br_{0.15})_3$ perovskite film. The PL count-rate maps are normalised to their respective mean, while in the PL count-rate histogram (g) each distribution is normalised to its mean. The lifetime and spectral median maps (b, c, e and f) are respectively plotted on the same scale for direct visual comparison. The maps are recorded nominally on the same scan region, though there is some error in the precise alignment. The maps show that the PL is more uniform and homogeneous at the surface than below (a, d and g), and that this homogeneity is accompanied by longer lifetimes (b, e and h) and a blueshift (c, f and i).

Passivated Samples:

A different $MAPbI_3$ film was used for comparative measurements with the treated film shown in Figure 2 of the main text. The histograms in Figure 3 of the main text were extracted from the PL count rate (Figure S8a and d), lifetime (Figure S8b and c) and spectral median (Figure S8c and f) maps recorded on this sample. The contrast between 1P and 2P excitation was weaker on this film than on the one shown in Figure S4 and S5, which may be due to some ageing, small atmospheric exposure in transit or a very slightly higher excitation fluence (such that carrier diffusion may influence the maps). The lifetime and spectral median do not appear to change at or below the surface.

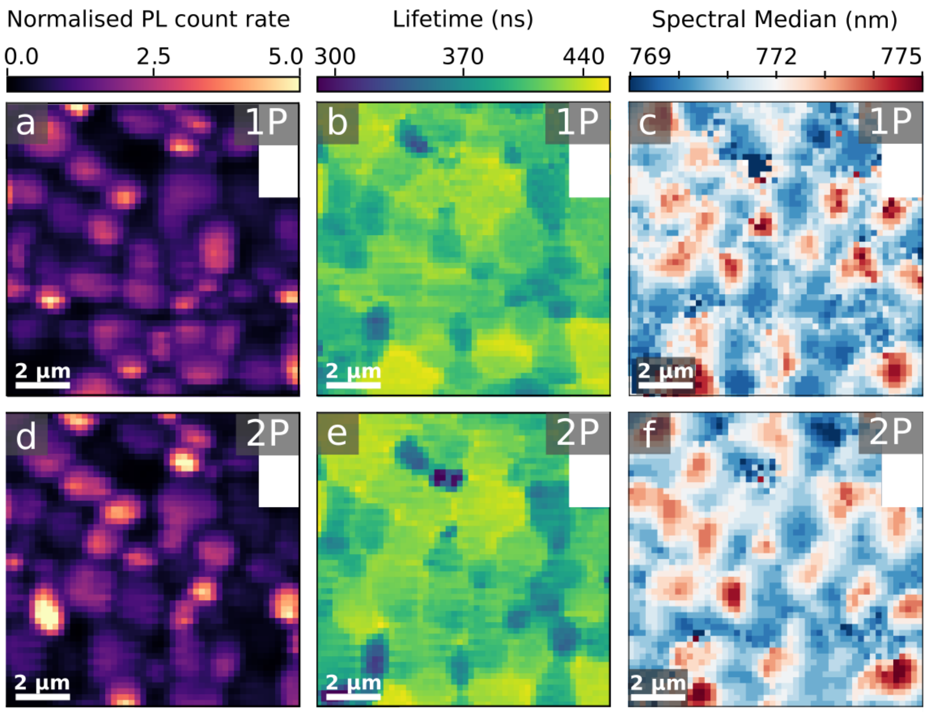

Figure S8. $MAPbI_3$ film. (a,d) PL count rate maps acquired using 1P and 2P excitation, normalised to their respective mean. (b,e) Lifetime maps extracted from 1P- and 2P-TRPL measurements. (c,f) Spectral median maps extracted from 1P- and 2P-PL spectra. Regions denoted in white enclose marker particles on the surface of the film, which were excluded from the correlation analyses.

In order to compare the luminescence of the untreated and treated films at and below the surface, the PL maps of the treated film in Figure S9a and d are normalised to the maximum obtained with 1P and 2P on the untreated film in Figure S8(a,d), respectively.

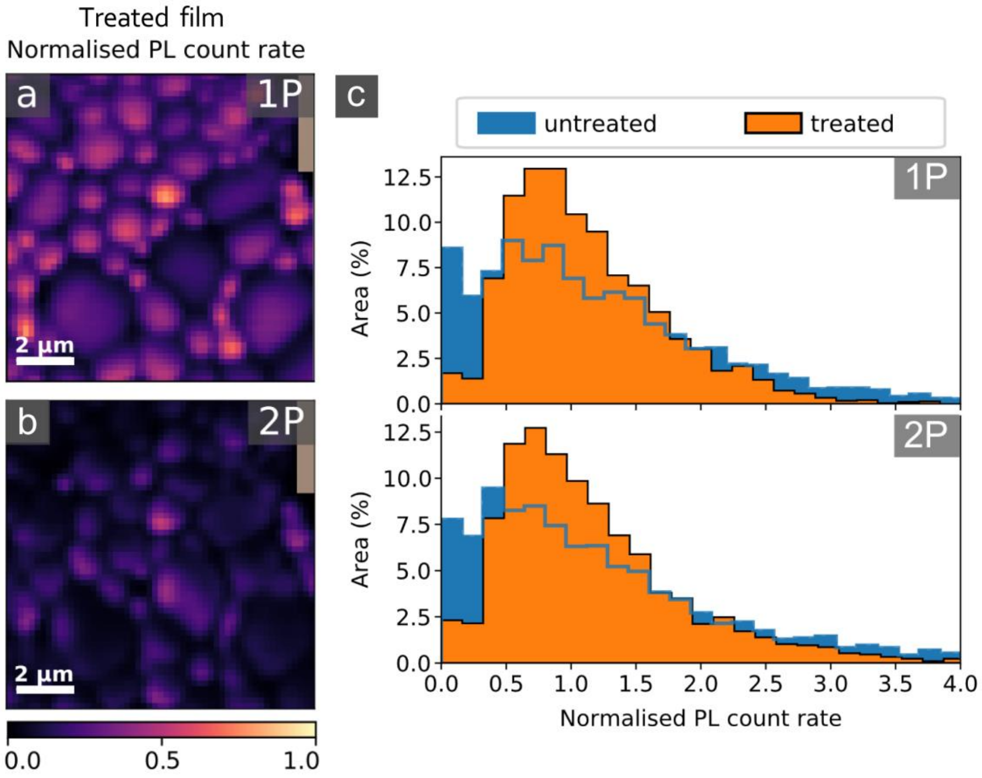

Figure S9. $MAPbI_3$ film after light soaking in humid air. (a,b) PL count rate maps acquired using 1P and 2P excitation. The 1P map in (a) is normalised to the maximum of its untreated counterpart in Figure S8a. In the same way, the 2P map in (d) is normalised to the maximum of Figure S8d. Shaded regions enclose marker particles on the surface of the film, excluded from the analysis. (c) Comparison between the PL count rates for 1P and 2P excitation on the untreated and treated films. Each distribution is normalised to its median value.

In order to assess potential correlations between the PL and lifetime for both films, untreated and treated, we show in Figure S10 scatter plots of the data contained in the maps (blue dots).

We binned the PL count rate data (into 15 bins) and computed the mean lifetime for each bin (orange dots). As a guide to the eye, we performed a linear fit on the binned data (red dotted lines). Bins containing fewer than 10 data points were discarded and excluded from the analysis. In the untreated sample, brighter areas appear to have a longer lifetime under both 1P (Figure S10a) and 2P (Figure S10c) excitation, whereas we observe the opposite trend in the treated sample, again with both 1P (Figure S10b) and 2P (Figure S10d) excitation.

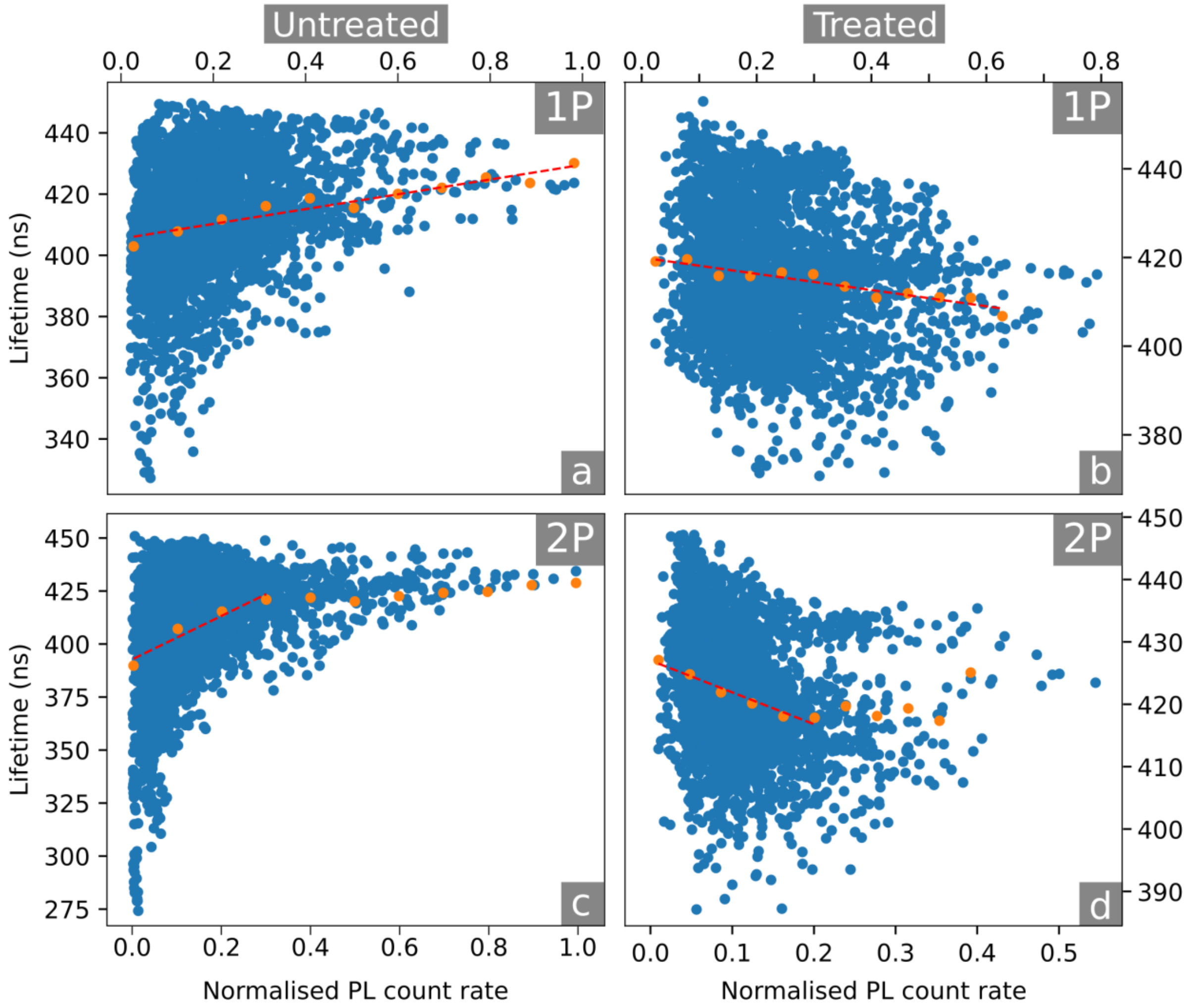

Figure S10. Spatial correlations between PL and lifetime in untreated (a, c) and treated (b, d) films, using one-photon (1P) and two-photon (2P) excitation.

We also considered correlations between the 1PPL and 2PPL maps, for both the untreated and treated samples (Figure S11). Grains that appear bright at the surface seem to be bright as well in the bulk. The scatter plot for the untreated film (Figure S11, left panel) contains fewer data points than for the treated film (Figure S11, right panel), because only the data contained in the top half of the map was used for the correlations. The bottom half of this 1PPL map (shown in Figure S8a appears distorted (shrunk) when compared with the 2PPL map in Figure S8d), and we therefore did not use it to evaluate spatial correlations.

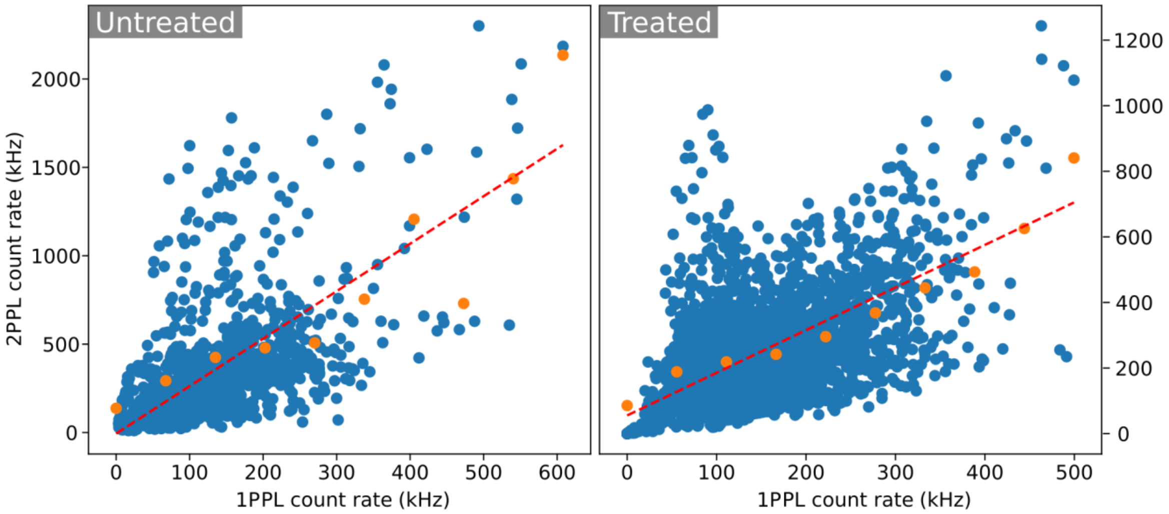

Figure S11. Spatial correlations between 1PPL and 2PPL in untreated (left) and treated (right) films, using one-photon (1P) and two-photon (2P) excitation, respectively.

Bulk Crystal Films:

We also performed 3D time-resolved tomography on a micro-crystal film of $MAPbI_3$ (XRD data shown in Figure S12). The chosen PL count-rate and lifetime isosurfaces are shown in Figure S13a, b and c, along with a 2P optical image of the luminescence of a single micro-crystal in Figure S13d.

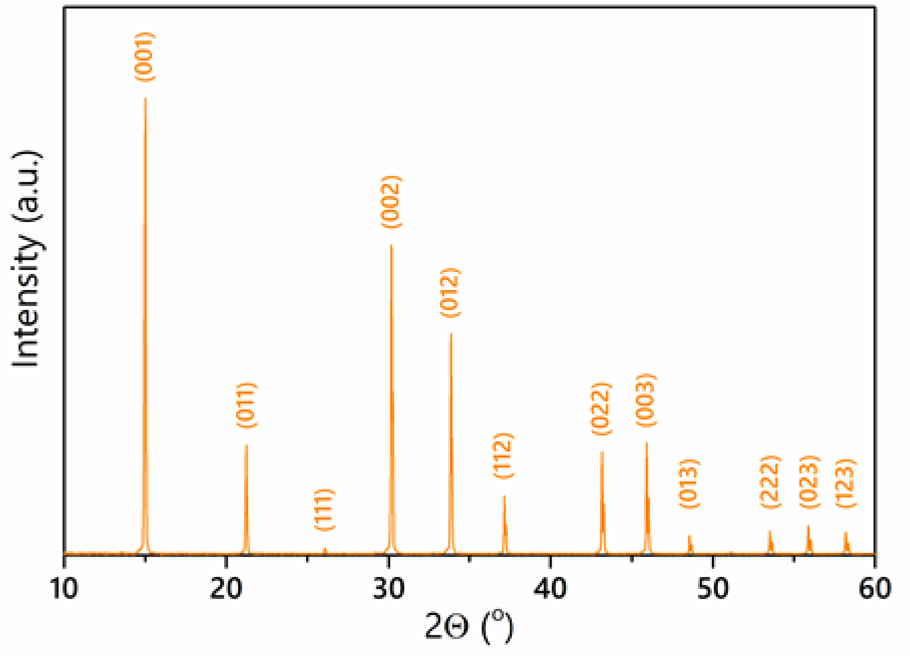

Figure S12. X-Ray Diffraction pattern measurement of a $MAPbBr_3$ micro-crystal film.

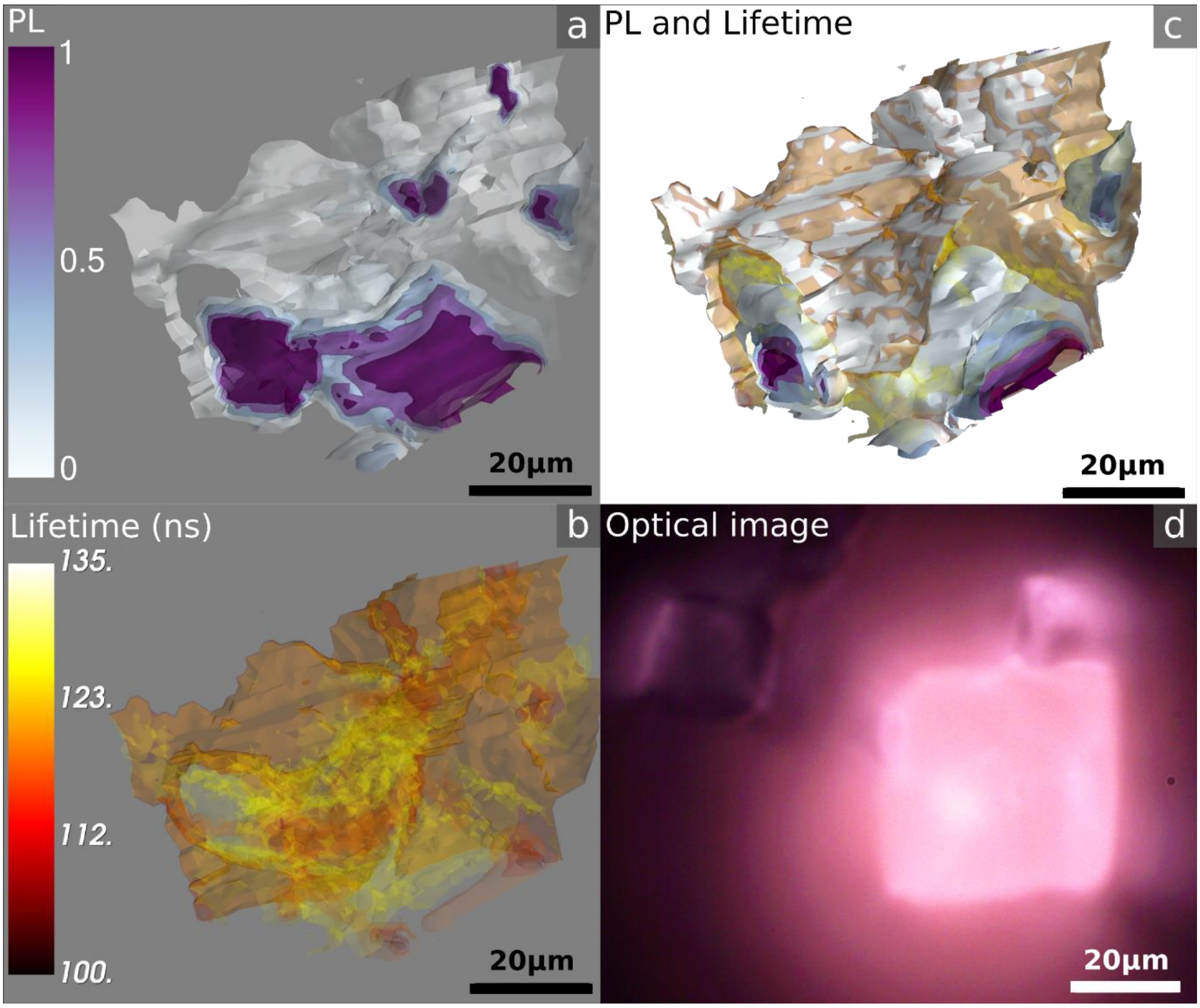

Figure S13. Time-resolved 3D tomography of a micro-crystal film of $MAPbI_3$ using 2P-TRPL at 1100 nm with a resolution of ~1 µm in depth. (a) The 3D count rate and (b) the extracted lifetime, which are both superimposed in (c). (d) Two-photon optical image of crystal from the top.

For a better and easier visualisation of the PL decay over time, the time resolved images in Figure 4a (main text) contain only the upper 3 quartiles of the relative PL count rate. For completeness, we show in Figure S14 the full data including measurement noise and wrap-around effects (excluding only pixels outside of the crystals), with a different projection than in Figure 4.

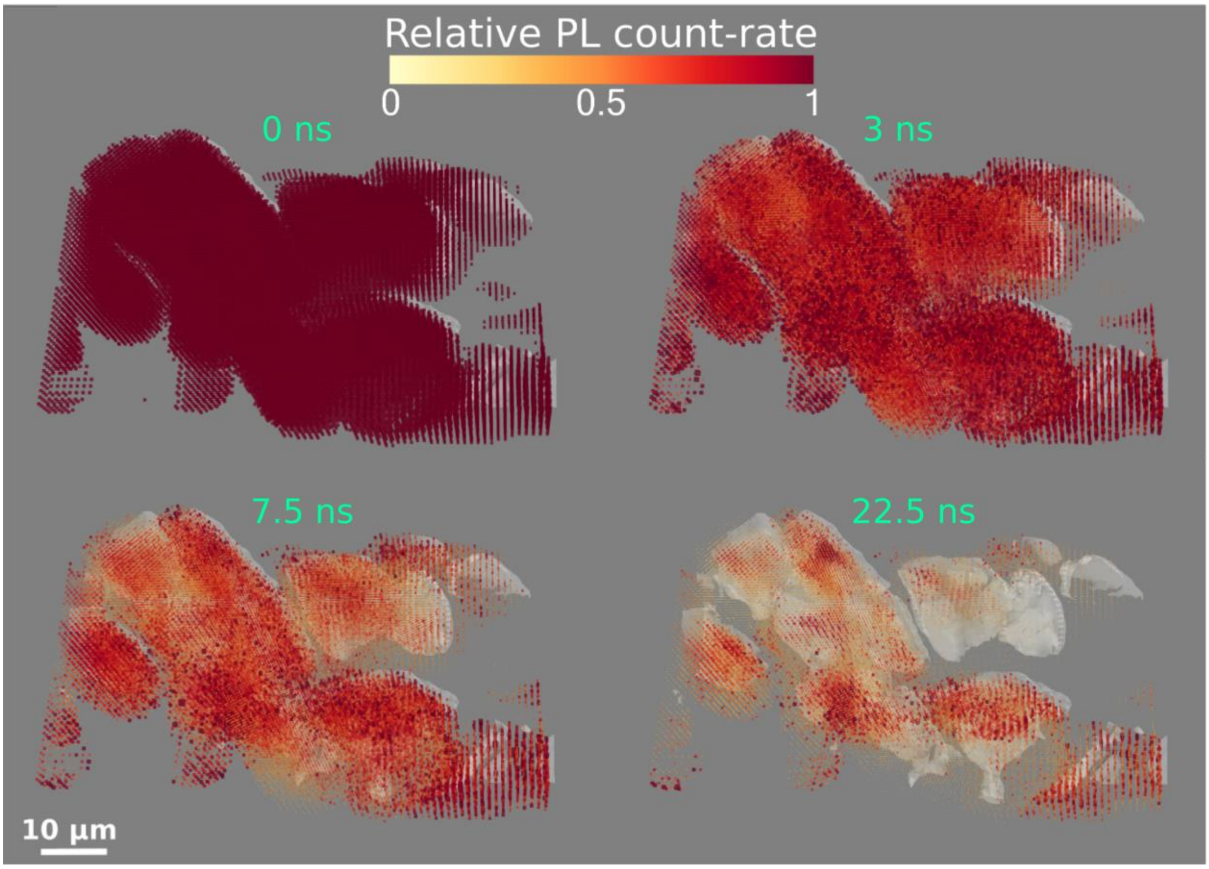

Figure S14. 3D time-resolved PL decay with a topography overlay of a micro-crystal film of $MAPbBr_3$ using 2P-TRPL at 1100 nm with a step size of 1 μm in depth. The full scale data is presented with only background pixels removed outside of the crystals.

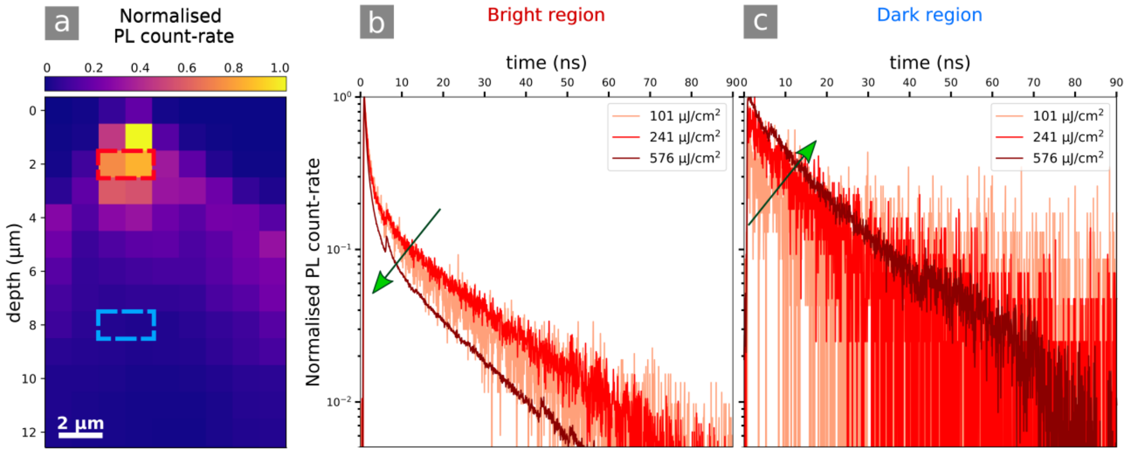

Figure S15. Fluence-dependent measurements on a micro-crystal film of $MAPbBr_3$ using 2P-TRPL with a resolution of ~1 µm in depth. The PL count-rate map in (a) was performed at a fluence of 232 µJ/cm^2. The panels in (b) and (c) show respectively TRPL measurements averaged over two adjacent pixels of comparable brightness, at 2 µm (b) and 8 µm (c) below the surface. Green arrows indicate the dependence of the lifetime upon an increase in excitation power, highlighting the different recombination regimes discussed in the text. All measurements are normalised to their maximum.

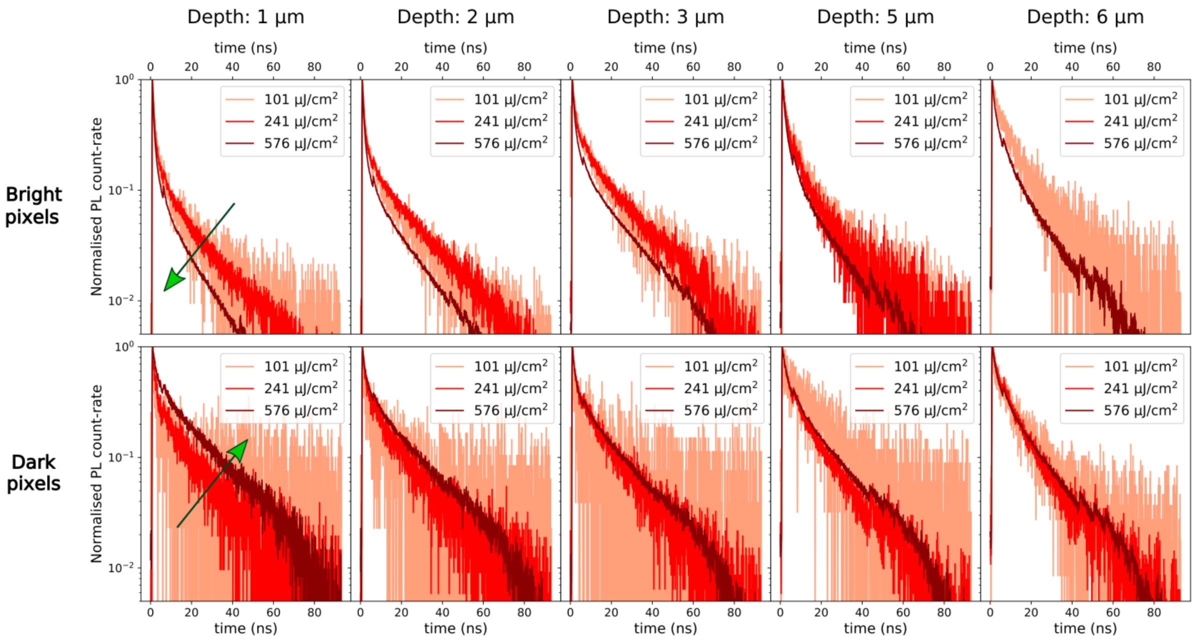

Figure S16. Fluence-dependent measurements on a micro-crystal film of $MAPbBr_3$ using 2P-TRPL with a resolution of ~1 µm in depth. Each column shows TRPL measurements taken at a given depth, from 1 µm to 6 µm, with pulse fluences as stated on the legends. The top and bottom rows highlight measurements performed on bright and dark pixels, respectively, averaged across all such pixels at each depth. Bright pixels are defined as pixels where the PL count-rate is above 30% of the maximum PL count-rate on the whole 2D map (shown in Figure S15a), while dark grains have a PL count-rate below 25% of this maximum. Green arrows in the left-most panel indicate the dependence of the lifetime upon an increase in excitation power, highlighting different recombination regimes. All measurements are normalised to their maximum.

References

1. Zhang, W. *et al.* Enhanced optoelectronic quality of perovskite thin films with hypophosphorous acid for planar heterojunction solar cells. *Nat. Commun.* **6,** (2015).

2. Brenes, R. *et al.* Metal Halide Perovskite Polycrystalline Films Exhibiting Properties of Single Crystals. *Joule* **1,** 155–167 (2017).

3. Abdi-Jalebi, M. *et al.* Impact of a Mesoporous Titania-Perovskite Interface on the Performance of Hybrid Organic-Inorganic Perovskite Solar Cells. *J. Phys. Chem. Lett.* **7,** 3264–3269 (2016).

4. Saidaminov, M. I. *et al.* High-quality bulk hybrid perovskite single crystals within minutes by inverse temperature crystallization. *Nat. Commun.* **6,** (2015).

5. Leguy, A. M. A. *et al.* Experimental and theoretical optical properties of methylammonium lead halide perovskites. *Nanoscale* **8,** 6317–6327 (2016).

6. Rumi, M. & Perry, J. W. Two-photon absorption: an overview of measurements and principles. *Adv. Opt. Photonics* **2,** 451 (2010).

7. Wei, Q. *et al.* Two-Photon Optical Properties in Individual Organic–Inorganic Perovskite Microplates. *Adv. Opt. Mater.* **5,** (2017).
